# NEIGHBORHOOD SEGREGATION AND CHARACTERISTICS OF OLDER CHINESE IMMIGRANTS LIVING IN GREATER CHICAGO

**DOI:** 10.1093/geroni/igad104.1408

**Published:** 2023-12-21

**Authors:** Yanping Jiang, Fengyan Tang, Dinesh Mendhe

**Affiliations:** Rutgers University, New Brunswick, New Jersey, United States; University of Pittsburgh, Pittsburgh, Pennsylvania, United States; Rutgers University New Brunswick, Piscataway, New Jersey, United States

## Abstract

Neighborhoods profoundly influence health of older adults, with those living in more affluent neighborhoods tending to be happier and healthier. However, which neighborhoods to live, is shaped by pull and push factors, particularly among racial/ethnic minorities that disproportionally live in segregated neighborhoods characterized by social and economic deprivation. Residential segregation, however, is not well-examined among older Asian Americans. This study aimed to close this gap by investigating neighborhood, sociodemographic, and health behavior characteristics among older Chinese immigrants living in segregated neighborhoods. Data were drawn from 3,097 participants from Population Study of Chinese Elderly (PINE). Residential segregation was operationalized using Index of Concentrations at the Extremes (ICE), and the tertiles of ICE scores were used to categorize neighborhoods into four groups, ranging from neighborhoods extremely segregated toward Chinese speakers to segregated toward English-only speakers. Results showed that, compared to neighborhoods segregated toward English-only speakers, neighborhoods extremely segregated toward Chinese speakers had lower neighborhood socioeconomic status and cohesion but higher neighborhood disorder. Also, participants in neighborhoods segregated toward Chinese speakers were more likely to be unmarried but less likely to live alone, receive less education, and live in the U.S. longer. These participants also reported lower acculturation and racial discrimination, were more likely to smoke, and had lower adoption of preventive health practices than those living in neighborhoods segregated with English-only speakers. Findings highlight challenges and opportunities among older Chinese immigrants living in segregated neighborhoods and provide insights into existing mixed findings on health impacts of residential segregation among this understudied population.

